# Lamellar Granule Secretion Starts before the Establishment of Tight Junction Barrier for Paracellular Tracers in Mammalian Epidermis

**DOI:** 10.1371/journal.pone.0031641

**Published:** 2012-02-06

**Authors:** Akemi Ishida-Yamamoto, Mari Kishibe, Masamoto Murakami, Masaru Honma, Hidetoshi Takahashi, Hajime Iizuka

**Affiliations:** Department of Dermatology, Asahikawa Medical University, Asahikawa, Japan; University of Birmingham, United Kingdom

## Abstract

Defects in epidermal barrier function and/or vesicular transport underlie severe skin diseases including ichthyosis and atopic dermatitis. Tight junctions (TJs) form a single layered network in simple epithelia. TJs are important for both barrier functions and vesicular transport. Epidermis is stratified epithelia and lamellar granules (LGs) are secreted from the stratum granulosum (SG) in a sequential manner. Previously, continuous TJs and paracellular permeability barriers were found in the second layer (SG2) of SG in mice, but their fate and correlation with LG secretion have been poorly understood. We studied epidermal TJ-related structures in humans and in mice and found occludin/ZO-1 immunoreactive multilayered networks spanning the first layer of SG (SG1) and SG2. Paracellular penetration tracer passed through some TJs in SG2, but not in SG1. LG secretion into the paracellular tracer positive spaces started below the level of TJs of SG1. Our study suggests that LG-secretion starts before the establishment of TJ barrier in the mammalian epidermis.

## Introduction

Tight junctions (TJs) are cell-cell junctions that connect neighboring cells closely [1], [2], [3]. One of the important functions of TJs is to provide a paracellular permeability barrier regulating the movement of water, solutes, and immune cells in the tissue. The most apical region of the lateral membranes of simple epithelial cells is linked together by junctional complexes that consist of TJs, adherens junctions, and desmosomes. Typical junctional complexes have not been detected in the epidermis which is a continuously renewing stratified epithelium covering the outer surface of the body. Although mutations in the gene encoding claudin, an integral component of TJs, result in severe barrier defects and ichthyosis [4], [5], disagreement exists as to the presence of TJs in the epidermis. In an EM study using an electron dense tracer lanthanum, Hashimoto suggested that they exist in the stratum granulosum (SG) [6]. Two other studies with a paracellular tracer on light microscopic level also supported this finding [2], [7]. However, a network of anastomosing fibrils, which is typical of TJ structure in freeze-fracture EM in simple epithelia, was not detected in the epidermis [8].

One of the major functions of the epidermis is to provide an inside-out permeability barrier to prevent dehydration. Two structures are thought to be involved in the paracellular permeability barrier function. One is the intercellular lipid layer of the stratum corneum (SC) [9]. The other one is TJs in the SG [2], [4], [6], [7], [10]. A recent study suggested that the most superficial layer of the SG (SG1) is devoid of TJs, but that the second layer (SG2) possesses TJ networks surrounding each cell in mouse epidermis [11]. The significance of the TJ-free nature of SG1 has been emphasized as being an intermediate layer that bridges air-liquid and liquid-liquid interface providing an environment for Langerhans cells to take antigens across TJ barriers. In that case, discontinuity exists between the TJ-based barrier in the SG and intercellular lipid-based barrier in the SC.

Another important function of TJs is to create and establish apical and basolateral membrane domains functioning as a “fence” while playing a role in vesicular transport. We have reported evidence suggesting that secretion of lamellar granules (LGs) is mediated by a SNARE-mediated vesicular fusion process [12], [13]. Our previous study also suggested that apical transportation and secretion of LGs are dependent upon functional TJs [14]. The above concept of TJs only being present in SG2 does not fit with the fact that most if not all LGs are secreted from the apical membrane domain of SG1. Previously, we found that LG-secretion is a sequential process which enables secretion of various cargos in different timing of epidermal differentiation [15]. We supposed that the epidermis might have its own unique TJ system adapted for sequential LG-secretion.

The aim of the present study is to determine overall structure of TJs in the epidermis in relation to LG-secretion and delineate barrier sites. We found evidence to suggest that the epidermal TJ-related proteins form hitherto unrecognized multilayered network structures. We propose that multilayered TJ-related protein immunoreactive structures enable the epidermis to uphold a robust permeability barrier while coping with continuous turnover of the cells as well as allowing sequential secretion of LGs.

## Materials and Methods

### Materials

Normal adult human skin was obtained during plastic surgery procedures. All participants gave informed written consent and the protocol was approved by the Medical Ethics Committee of the Asahikawa Medical University. The study was conducted according to the principles of the Helsinki declaration. Hairless mice (HOS:hr-1) were purchased from Sankyo Labo Service Corporation (Tokyo, Japan). All of the experimental protocols were carried out according to the protocols approved by the Institutional Animal Care and Use Committee of Asahikawa Medical University (permit No. 11053).

### Antibodies

Antibodies and dilutions used were as follows: 3G217 (1∶100, anti-occludin C-terminus; mouse, Lifespan BioSciences, Seattle, WA), 71-1500 (1∶50, anti-occludin C-terminus; rabbit, Zymed, South San Francisco, CA), ZO-1 (1∶100, rabbit, Zymed), CDSN (1∶250, anti-CDSN central domain; rabbit; Ishida-Yamamoto Laboratory) [16], and GlcCer (1∶1000, rabbit, Glycobiotech, KüKels, Germany). Preimmune rabbit serum and control mouse-IgG (DAKO, Glostrup, Denmark) were used as negative controls. Secondary reagents used for immunofluorescence were goat anti-mouse, and anti-rabbit IgG linked to Alexa-Fluor 488 (1∶100, Molecular Probes, Eugene, OR), and Cy3-labeled goat anti-rabbit IgG (1∶20, Amersham Bioscience, Buckinghamshire, UK). The secondary antibodies used for immunoelectron microscopy were 5-nm or 10-nm gold-conjugated goat anti-rabbit IgG (1∶50, Amersham Bioscience), or Nanogold® gold anti-mouse IgG (1∶10, Nanoprobes, Yaphank, NY) intensified with HQ Silver® Enhancement Kit (Nanoprobes).

### Assessment of permeability by a biotin tracer

Permeability assay using a surface biotinylation technique was performed using dermal injection of 100 µl of 10 mg/ml EZ-Link® Sulfo-NHS-LC-Biotin (Pierce Biotechnology, Rockford, IL) on excised normal human skin tissue samples (n = 3) cultivated with a high calcium (1.2 mM) medium (CnT-02-3DP1, CellnTEC, Bern, Switzerland) at an air-medium interface of Cell Culture Insert (Becton Dickinson, Franklin Lakes, NJ) at 37°C. The skin tissue was fixed 30 min after injection with 2% paraformaldehyde for 1 h at 4°C and treated with 100 mM glycine in PBS (pH 7.4) for 1 h at 4°C. For fluorescent microscopy, biotin was detected with avidin conjugates-linked to Alexa Fluor 488 (1∶500, Molecular Probes, Eugene, OR). For EM, biotin was detected with EM Streptavidin/Gold, 10 nm (1∶200, Electron Microscopy Sciences, Hatfield, PA) and intensified with HQ Silver® Enhancement Kit (Nanoprobes).

### Assessment of permeability by lanthanum perfusion

Freshly obtained biopsies were exposed to 4% colloidal lanthanum nitrate tracer in 0.05 M Tris buffer, pH7.4, containing 2% glutaraldehyde and 2% paraformaldehyde at RT for 1 h. The samples were then washed and processed for EM as described below. To measure LG-areas, electron micrographs were taken at a magnification of 20,000 and their montages were prepared using Adobe Photoshop CS4 (Adobe Systems, San Jose, CA). LG-areas were measured using Image-Pro Plus ver. 4.0 imaging software (Media Cybernetics, Silver Spring, MD). Randomly chosen 5 ultrathin human sections (n = 2) and 11 mouse sections (n = 4) were analyzed, and the data were expressed as means ± SEM.

### Immunofluorescent microscopy

Unfixed or biotin tracer injected and fixed human skin tissue were embedded in O.C.T. compound (Sakura Finetechnica, Tokyo, Japan) and frozen. Three-micrometer-thick cryostat sections were mounted on slides. The sections were fixed with acetone at −30°C for 10 min, pre-incubated with 10% normal goat serum (NGS) in phosphate buffered saline (PBS) for 15 min at RT and permeabilized in 0.3% Triton X-100 in PBS for 5 min at RT. They were incubated with primary antibodies diluted in 0.3% Triton X-100, 1% NGS, 1% bovine serum albumin in PBS (incubation buffer) for 30 min at 37°C. After washing in PBS, they were incubated with fluorescent secondary antibodies diluted in PBS for 30 min at 37°C. Nuclei were stained with 4′-6-diamidine-2-phenyl indole (DAPI) and mounted on slides with an aqueous-based mounting medium (PermaFluor, Thermo Fisher Scientific, Waltham, MA).

Biopsies from various body sites (one biopsy for each site) of human and mouse were incubated with 20 mM EDTA in PBS for 1–3 h at 37°C and epidermal sheets were separated from the dermis. The sheets were fixed with 2% paraformaldehyde in PBS for 1 h at 4°C and treated with 100 mM glycine in PBS for 1 h at 4°C. They were further fixed with acetone for 10 min at −30°C. They were pre-incubated with 10% NGS in PBS for 15 min at RT, and incubation buffer for 5 min at RT. They were incubated with primary antibodies diluted in incubation buffer for 2 d at 4°C. After washing in PBS, they were incubated with fluorescent secondary antibodies diluted in the same incubation buffer for 2 d at 4°C. Nuclei were stained with propidium iodide (PI) and mounted on slides with an aqueous-based mounting medium.

Fluorescent images (single optical slices or up to 64 multiple slices Z-spacing of 0.43 µm) were captured at RT using a Fluoview FV1000-D microscope equipped with a BX61 microscopy (Olympus, Tokyo, Japan) fitted with a 100×/1.40 UPlan-SApo oil objective lens (Plan-Apochromat) and analyzed using Olympus FV1000 software FV10-ASW version 2.1.

### EM and Immunoelectron microscopy

EM and post-embedding immunoelectron microscopy using Lowicryl K11M resin (Chemische Werke Lowi)-embedded skin samples was performed as described previously [15], [17].

## Results

### Epidermal tight junction-related proteins form multilayered networks

**Figure 1:**
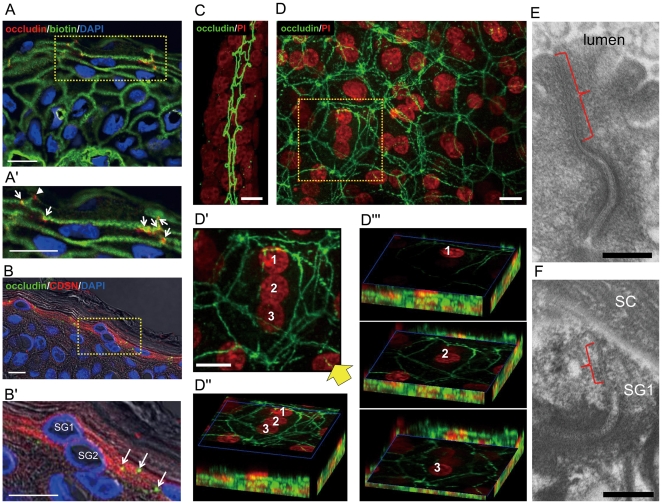
Multilayered TJ-protein immunoreactive networks in the human epidermis. (A, A′) Biotin-tracer passed through some occludin-positive dots (arrows), but stopped at the most superficial dots (arrowhead). (B, B′) The CDSN-positive SG1 and SG2 have occludin-positive dots (arrows). (C, D) Occludin immunostaining of a sweat duct (C) and an epidermal sheet (D). D and D″ show a projection image of the upper three nucleated cell layers. D″ shows a 3D image from the corner (arrow). D′″ shows slice views dissecting uppermost (1), the second (2) and the third (3) cells. (E, F) EM of TJs (brackets) in the sweat duct (E) and the epidermis (F). A′, B′ and D′ are high-magnification views of A, B and D respectively. Bars = 10 µm (A–D′) and 200 nm (E, F).

In order to reveal the true nature of the TJ-related structures in the human epidermis, we studied the expression of occludin, a major transmembrane component of TJs, in vertical skin sections and in the epidermal sheet. In the vertical sections, occludin-positive dots were detected at the cellular margins in the upper two to three layers of the SG (Fig. 1A and B). To correlate paracellular permeability and occludin localization, we injected a protein biotinylation reagent into the dermis. The tracer passed through one or two occludin-dots and stopped at the uppermost dots suggesting leakiness of the TJ-related structures in the lower layers (Fig. 1A) as has been reported by others [2]. Because TJs are also implicated in vesicular transport, we compared expression of occludin and corneodesmosin (CDSN), a secretory protein transported and secreted by LGs [18]. The upper two to three layers of SG were CDSN-positive and the staining was strong at the apical side of the cells (Fig. 1B). These cells expressed occludin at the cellular margins as well.

To verify that multiple cell layers express occludin, we observed its expression in whole mount epidermal sheets. Adequacy of the tissue preparation and immunostaining methods was verified by well-preserved occludin-positive TJ-networks in the eccrine sweat ducts which were simultaneously peeled off with the epidermis from dermal connective tissues (Fig. 1C). In the epidermal sheets, occludin staining showed branching thin lines forming multilayered network structures spanning SG1 to SG2 or the third layer (SG3) of the SG (Fig. 1D). Occludin-positive lines entirely or partially encircle each cell. This multilayered pattern was constantly observed in the epidermal sheets obtained from different body sites (forehead, retro-auricular area, and buttocks). Similar multilayered networks were observed in newborn and adult mouse epidermal sheets obtained from various locations (ears, chin, back, abdomen, dorsal feet) stained with a ZO-1 antibody (Supplementary Fig. S1).

**Figure 2:**
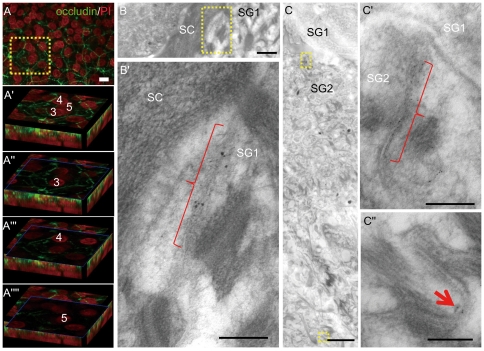
Occludin-labels are associated with TJ and non-TJ structures. (A) A projection image of deeper layers of the same area of the epidermal sheet shown in Figure 1D. (A′-A″″) 3D images of the third (3), fourth (4) and fifth (5) cells from the uppermost nucleated cell layers of the square in A. (B, C) Occludin immunoelectron microscopy. The labels are associated with TJs (brackets) in SG1 and SG2, but with small dots on the cell membrane in the lower epidermis (C″) (arrow). Silver intensified nanogold labels (B) and 5 nm-sized gold labels (C). B′, C′ and C″ are high-magnification views of B and C. Bars = 10 µm (A), 500 nm (B, C) and 200 nm (B′, C′, and C″).

**Figure 3:**
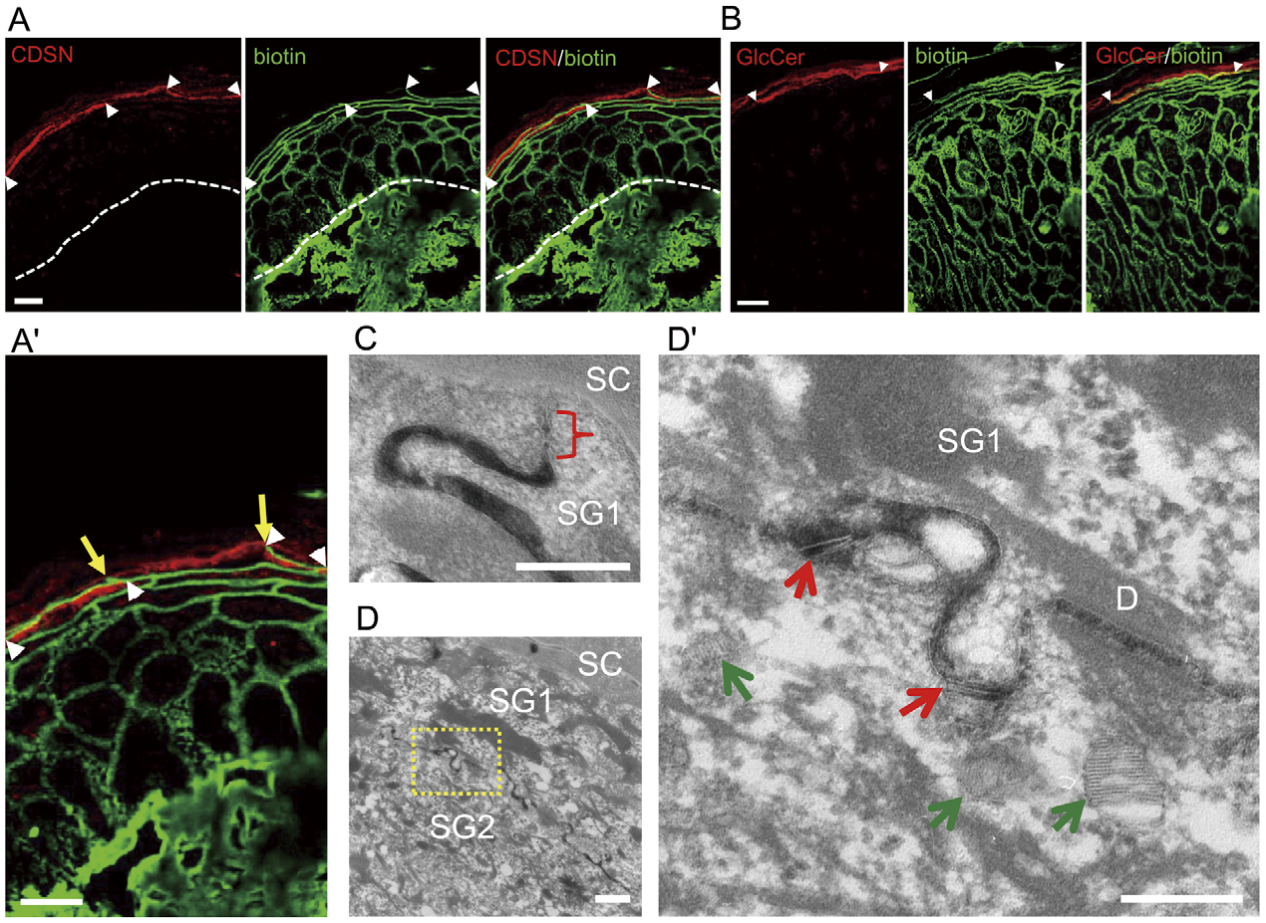
LGs start being secreted before establishment of paracellular permeability barrier. (A, B) TJ permeability assay using a surface biotinylation technique. LG molecules, CDSN and glucosylceramides (GlcCer) are co-localized with biotin (areas between arrowheads). A part of CDSN/biotin image in A is magnified in A′ and the points where the tracer stops are indicated with yellow arrows. The basement membrane zone is highlighted by the broken line. (C, D) Lanthanum penetration assay on normal human skin. (C) Lanthanum penetration is blocked at the TJ (bracket) in the SG1. (D) Secreted LGs (red arrows) can be found in the tracer permeable intercellular space. Green arrows indicate LGs in the cytoplasm. The area in the rectangle with broken lines in D is magnified in D′. D, desmosome. Bars = 10 µm (A, A′, B), 200 nm (C, D′) and 500 nm (D).

We also noted that the epidermal occludin-positive lines were thinner than those in the eccrine ducts (Fig. 1C and D). To verify this, we compared TJs of the epidermis with those of dermal eccrine sweat ducts at the ultrastructural level. TJs were indeed much smaller in the epidermis than in the sweat ducts (Fig. 1E and F). As to the level of TJs in the epidermis, we consistently found TJs in SG1 (Fig. 1F, 2B, 3C, Supplementary Fig. S2, S3A′, S3B′, Supplementary Fig. S4) as well as in SG2 both in humans and in mice.

### Occludin is associated with TJs in the upper granular cells and with non-TJ membrane domains in the lower epidermis

Because we found some occludin immunostaining in the layers lower than SG2 (Fig. 1D′″, 2A - A′″) and no TJ-like structures were found in these layers, we performed immunoelectron microscopy to clarify the nature of occludin-positive structures. In SG1 and SG2, immunoreactivities were associated with TJ-like structures (Fig. 2B, 2B′, 2C, 2C′, Supplementary Fig. S4). In the lower cells, it was associated with the plasma membrane domains not forming apparent junctional structures (Fig. 2C″), confirming previous observations by others [19].

### Lamellar granules start being secreted below the TJs in SG1

Because we found that epidermal TJ-related protein-immunoreactive structures are multilayered, we hypothesized that LGs start being released before the establishment of the final barrier for paracellular tracers. To correlate the timing of LG secretion and permeability barrier formation, we injected a biotin-tracer into the dermis and double-stained these sections with two LG-molecules, CDSN and glucosylceramides [15] (Fig. 3A and B). These LG-molecules first appeared intracellularly in the lower granular cells, and its staining pattern became linear at the apical side of upper granular cells. Merged images of the biotin-tracer and LG-molecules suggested their co-localization exists in a linear fashion. This suggests that LGs are secreted into the biotin-marker permeable extracellular spaces before the establishment of paracellular barrier. To verify these light microscopic observations with limited resolution, the following electron microscopy studies were performed.

**Figure 4:**
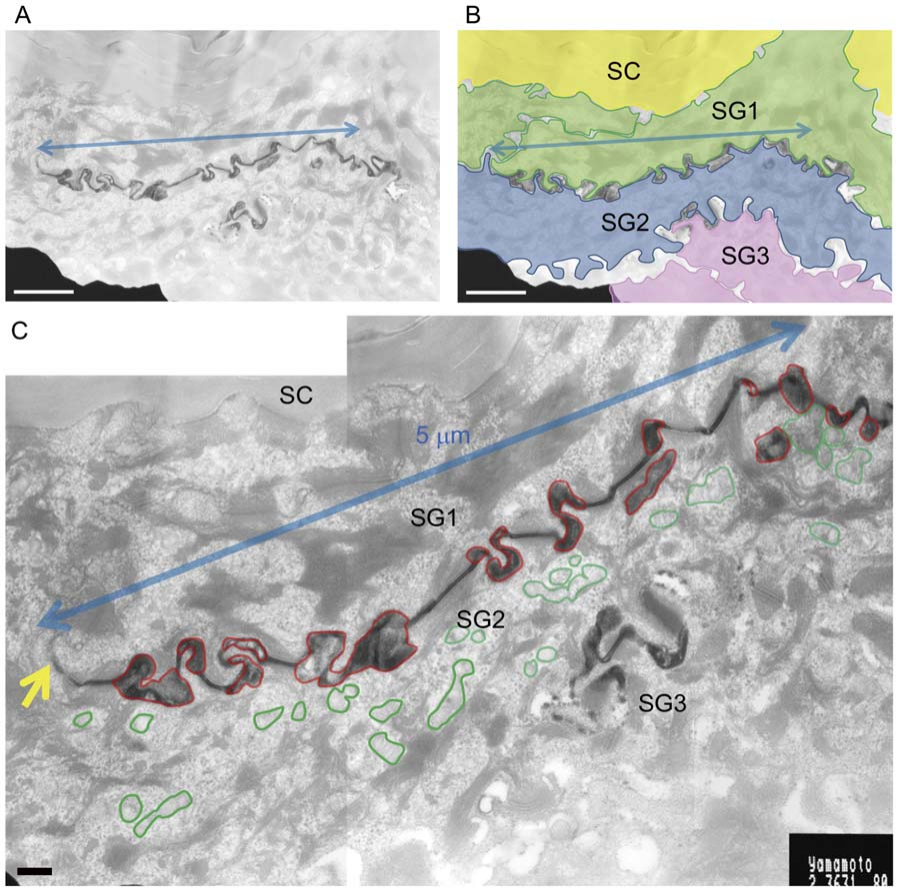
Secreted LGs are frequently found in the lanthanum-positive spaces. Lower (A, B) and higher (C) magnification views of a lanthanum penetration assay performed on normal human skin. LGs secreted into the lanthanum-positive extracellular spaces are outlined in red and those in the cytoplasm are outlined in green. In the 5 µm-width (blue arrows) area of a SG2 cell, secreted LG-areas are 0.510 µm^2^ and cytoplasmic ones are 0.376 µm^2^. The point where lanthanum stops is indicated with a yellow arrow. Lanthanum is lost during tissue preparation in the dilated intercellular spaces in the layers below SG3.

Morphological evidence to suggest LG-secretion occurring before paracellular barrier completion was also obtained from EM studies where secreted LGs were found at a level below that of the TJs in SG1 (Supplementary Fig. S2). To further confirm this, the inside-out movement of the low-molecular-weight, water-soluble, electron-dense tracer, colloidal lanthanum nitrate was used to assess permeability barrier establishment at the EM level. Penetration was blocked at TJs at the SG1 or SG2 level both in human and mouse epidermis (Fig. 3C, Supplementary Fig. S3A, and S3B). Fusion of LG membranes with cell membranes was found mainly above the level of cells with no intercellular lanthanum, but a certain proportion of LGs were found in the lanthanum-positive intercellular spaces (Fig. 3D, Supplementary Fig. 3C). All LGs secreted from SG1 are in lanthanum-free spaces beneath the SC. The extracellular spaces above the SG2 can be either lanthanum-positive or -negative probably depending on the tightness of TJs at each SG2 cell and on the effectiveness of tracer penetration into the tissue. The cells with leaky TJs may be underdeveloped. Because lanthanum penetration degrees varied greatly among different specimens (possibly also influenced by the sizes of the tissue blocks), quantitative analysis of lanthanum-positivity above SG2 was not performed in this study. We instead measured areas of LGs secreted into the lanthanum-positive extracellular spaces (areas which contained laminated or vesicular structures similar to those found in cytoplasmic LGs) and those of total (cytoplasmic and extracellular) LGs in the SG2 with lanthanum-positivity above the cells in each area of 5 µm widths. LG-areas in the lanthanum-positive spaces were 55.0±1.3% (n = 5) and 46.9±5.8% (n = 11) of the total LG-areas in the human and mouse epidermis, respectively (Fig. 4). LG secretion was only rarely found in SG3.

**Figure 5:**
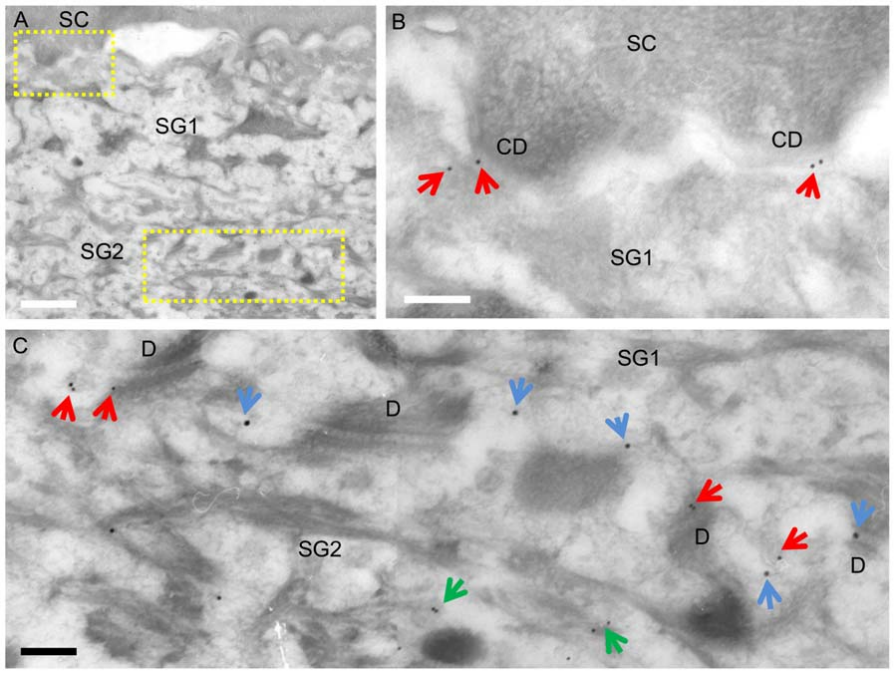
CDSN is found in the intercellular space (ICS) where a biotin tracer is detected. This figure is the result of the paracellular permeability assay using a biotin tracer performed on normal human skin. The areas in the rectangles in panel A are magnified in panels B and C. CDNS labels (red arrows) are seen in the biotin tracer negative ICS between SC and SG1 associating with corneodesmosomes (CD) (B), but they are in the tracer (blue arrows) positive ICS between SG1 and SG2 (C). Larger dots are silver intensified streptavidin/gold particles reacted with biotin. Smaller dots are 10 nm gold particles labelling CDSN. CDSN labels associated with LGs in the cytoplasm are indicated with green arrows. D, desmosome. Bars = 1 µm (A) and 200 nm (B, C).

Paracellular permeability analysis at the ultrastructural level was also performed using a biotin-tracer. The tracer was injected into the dermis and processed for immunoelectron microscopy. The biotin was detected using avidin-conjugated gold which was visualized using a silver intensification method. Double staining with occludin demonstrated that the tracer penetrated up to SG1 (Supplementary Fig. S4). No tracer was detected above the level of occludin-positive TJs in the most apical region of the lateral membrane of SG1. Double staining with CDSN revealed colocalization with the tracer at the intercellular spaces (Fig. 5). As to polarized transport of LGs in the cells, an EM study on more than 5 cells in each stratum of SG1, SG2 and SG3 showed that it was started by SG3 (Supplementary Fig. S5). Namely LGs were found from the middle to the apical side of the cells, but not in the basal side.

## Discussion

In the present study, we found occludin/ZO-1 immunoreactive multilayered networks spanning SG1 and SG2, differing from recent studies suggesting a single layered TJ-network in SG2 [4], [11]. Ultrastructurally typical TJs have been reported to exist in the SG1 [6], [20], [21]. Expression of occludin at the most superficial zone of the SG has also been reported [10]. Occasional two-layered occludin-positive network structures have also been noted in thick tangential sections of mouse epidermis [4]. Leakiness of lower epidermal TJs has been reported by Kirscher *et al*. [2] and was confirmed by our present study (Fig. 1A, Fig. S3B). Because of small sizes (Fig. 1F) and leakiness of epidermal TJs, multiplication of thin junctional structures may be needed to construct a robust permeability barrier. Recently, we found TJ-derived cell-cell contacts at the SC as well [17]. We consider that TJs, once assembled in SG2, remain thereafter to secure barrier function and hold barrier continuity between the SG and SC.

Because the molecular contents of LGs change after secretion, the rate of LG-areas in the lanthanum-positive spaces in total LG-areas obtained in the present study may not reflect the exact ratio of LG secretion. Nevertheless, the present study revealed that polarized transport (Fig. S5) and secretion (Fig. 3, Fig. 4, Fig. 5, Fig. S3) of LGs starts before establishment of a paracellular penetration barrier. LGs in lanthanum permeable intercellular spaces have been reported in a previous study as well [6]. A recent study showed a loss of polarization of LG secretion and a delay of TJ maturation/cell polarization in CD44 deficient mice [22]. In a previous study, we reported sodium caprate which disrupts TJs perturbed polarized secretion of LGs in cultured normal human keratinocytes and in a reconstructed human epidermis [14]. Combined with the present results, it is suggested that polarized transportation and secretion of LGs are not dependent upon establishment of intercellular tracer permeability barrier function of TJs, but may be dependent upon expression of TJ-related molecules at the cell membrane.

In the simple epithelia, redistribution of TJ molecules along the lateral plasma membrane sustains epithelial barrier function during physiological cell renewal [23]. Epidermis is stratified epithelia where cells continuously die and become cornified cells. The present results showed that the epidermis embraces a multilayered branching network of TJ-related protein-immunoreactive structures. This system adapts well to the continuous loss of living cells in cornification and is able to maintain TJ-related connections between neighboring granular cells. Our present study suggests that occludin first appears at the plasma membrane not associating with junctional structures (Fig. 2C″). Langbein *et al*. have also described various occludin-positive structures which are not typical TJs in mammalian stratified epithelia [19]. These structures may represent an early stage of TJ-related-network formation, but this should be tested in future studies.

All together, our findings increase the structural understanding of the skin barrier formation process. Because it has recently become clear that genetic defects or polymorphism of TJ components are associated with ichthyosis [5] and atopic dermatitis [24], and the expression/distribution of TJ proteins is abnormal in psoriasis [25], [26], further understanding of the whole TJ system in the skin is necessary to develop better methods to prevent or treat these skin diseases.

## Supporting Information

Figure S1 ZO-1 staining shows multilayered network in the mouse epidermis. ZO-1 staining (green) in the newborn mouse epidermal sheet from dorsal foot tissue. Nuclei were stained with PI (red). Bar = 10 µm.
(TIF)

Figure S2 EM features indicating LG secretion below the level of a TJ at the SG1. EM of normal human skin. The area in the rectangle on the left is magnified on the right. Bracket, TJ. Arrow, LGs found in the intercellular space. Bars = 200 nm.
(TIF)

Figure S3 Lanthanum is blocked at the SG1 or SG2 layer and LGs can be secreted into the tracer-permeable intercellular space. A lanthanum penetration assay on newborn mouse skin. (A, B) Lanthanum is blocked at a TJ (brackets) in SG2 (A″) or passed another TJ in SG2 (B″), but stopped at a TJ in SG1 (B′). The lower border of a cell in SG1 is highlighted by a dotted line in A. (C) Most LGs are secreted from the apical domain of the SG1 (green arrows). Some were secreted into the lanthanum containing intercellular spaces (red arrows). A′, A″, B′, B″, C′ and C″ are high-magnification views of A, B, C and C′. Bars = 1 µm (A, B, C) and 200 nm (the others).
(TIF)

Figure S4 A biotin tracer is detected below the level of occludin-positive TJs in SG1. This figure is the result of the paracellular permeability assay using a biotin tracer performed on normal human skin. (A) Larger dots are silver intensified streptavidin/gold particles reacted with biotin (blue circles). The tracer is detected up to SG1. (A′) The same field as in panel A, but each stratum is indicated with different colours. B, C and D are high-magnification views of A as indicated. E is a high-magnification view of D. Occludin-positive TJs are marked with red brackets. (F) The tracer is detected below the level of occludin-positive TJ in SG1, but not in the level above it. G is a high-magnification view of F. Bars = 2 µm (A, A′), 200 nm (B, C, E, G) and 500 nm (D, F).
(TIF)

Figure S5 EM features indicating polarized LG localization in the SG1, SG2 and SG3. EM of normal human skin. The middle and the right columns are high-magnification views of the left column showing the apical and basal parts of the cells, respectively. Arrows, LGs. The cell borders are highlighted by dotted lines. Bars = 1 µm (A, B, C) and 200 nm (the others).
(JPG)
