# Supplementary figures and images for: Lamellar Granule Secretion Starts before the Establishment of Tight Junction Barrier for Paracellular Tracers in Mammalian Epidermis
Source: PLoS One. 2012 Feb 6;7(2):e31641. doi: 10.1371/journal.pone.0031641 (PMC3273471; doi:10.1371/journal.pone.0031641)

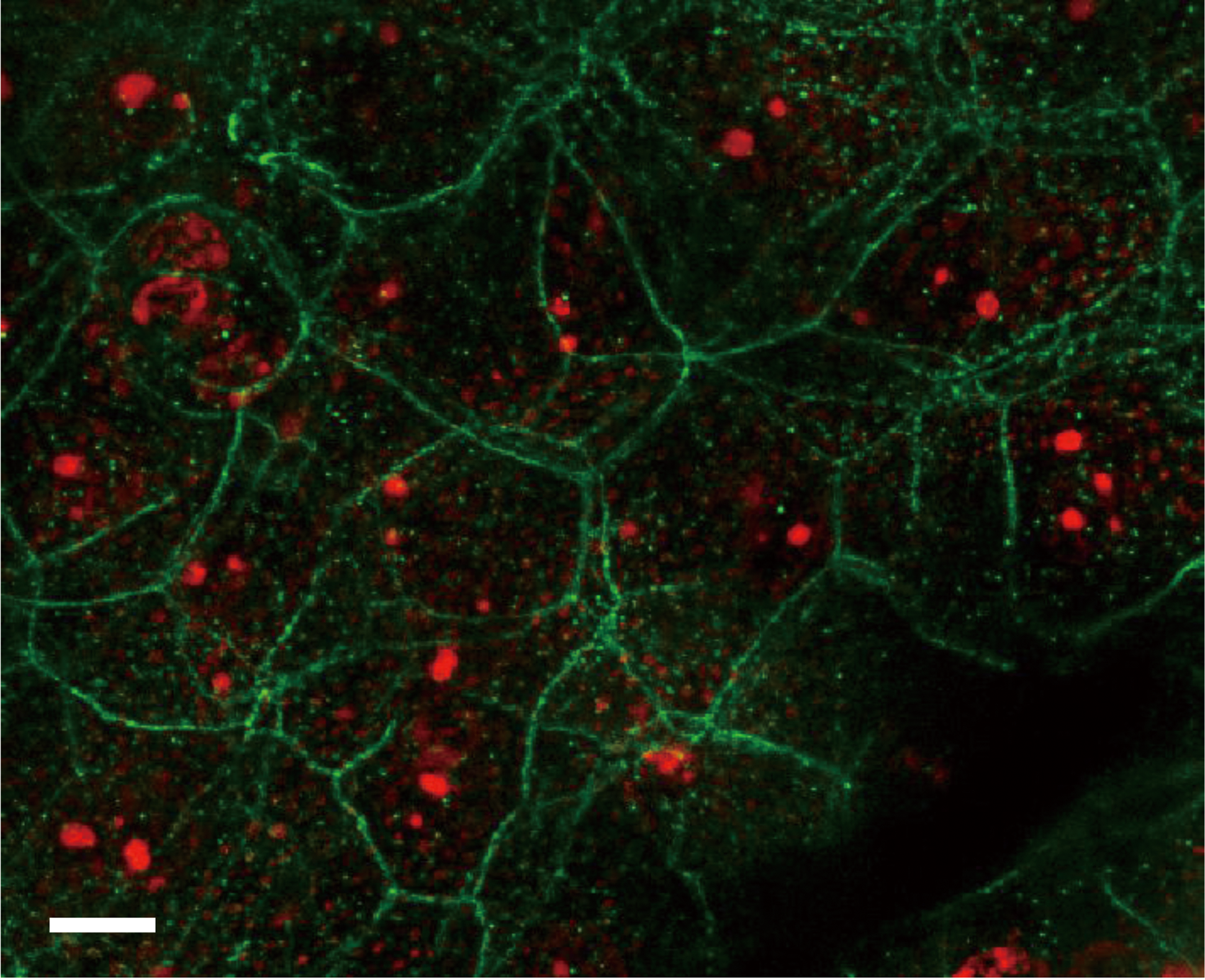

Supplement: Figure S1 — ZO-1 staining shows multilayered network in the mouse epidermis. ZO-1 staining (green) in the newborn mouse epidermal sheet from dorsal foot tissue. Nuclei were stained with PI (red). Bar = 10 µm. (TIF) [file pone.0031641.s001.tif]

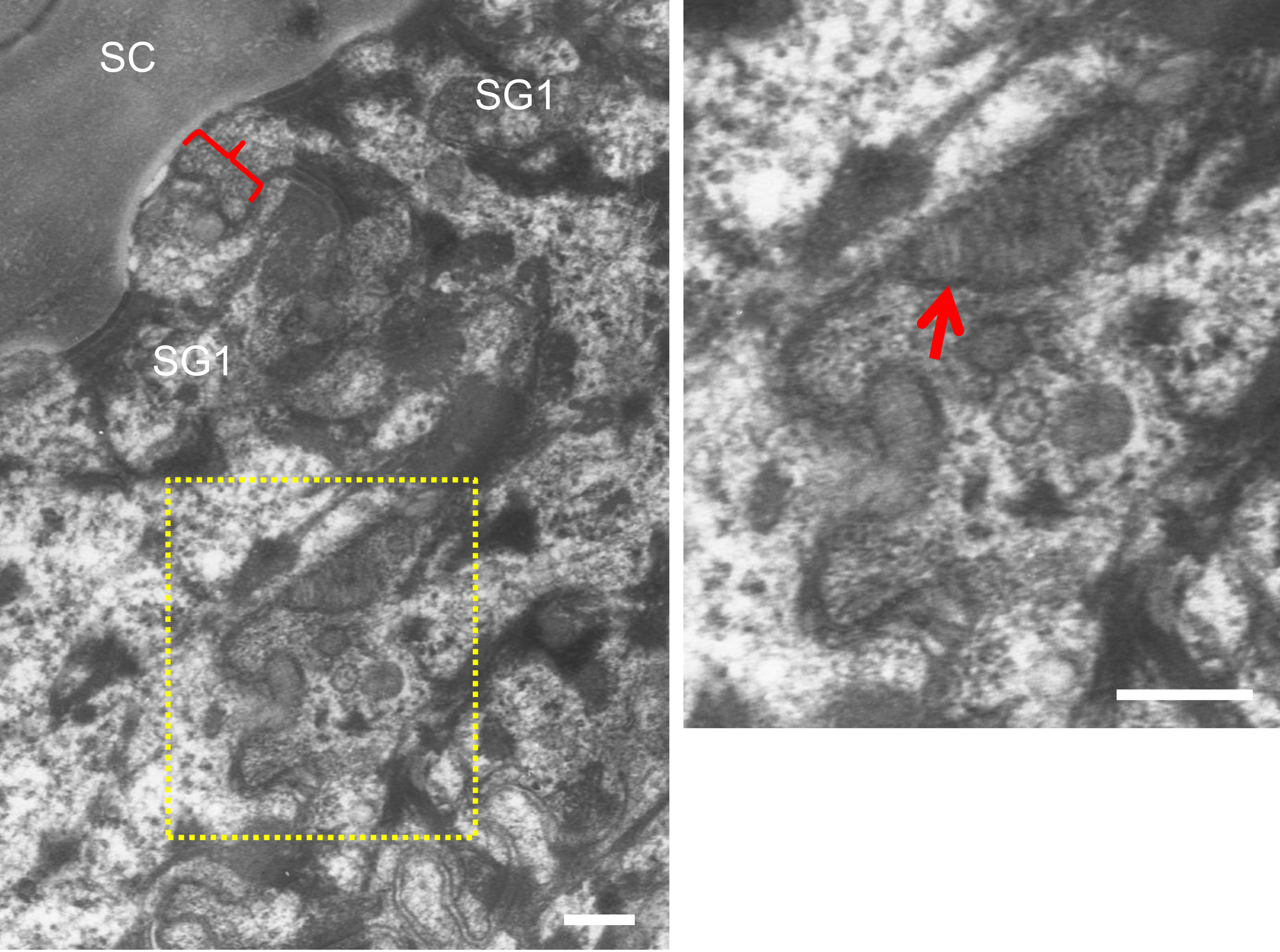

Supplement: Figure S2 — EM features indicating LG secretion below the level of a TJ at the SG1. EM of normal human skin. The area in the rectangle on the left is magnified on the right. Bracket, TJ. Arrow, LGs found in the intercellular space. Bars = 200 nm. (TIF) [file pone.0031641.s002.tif]

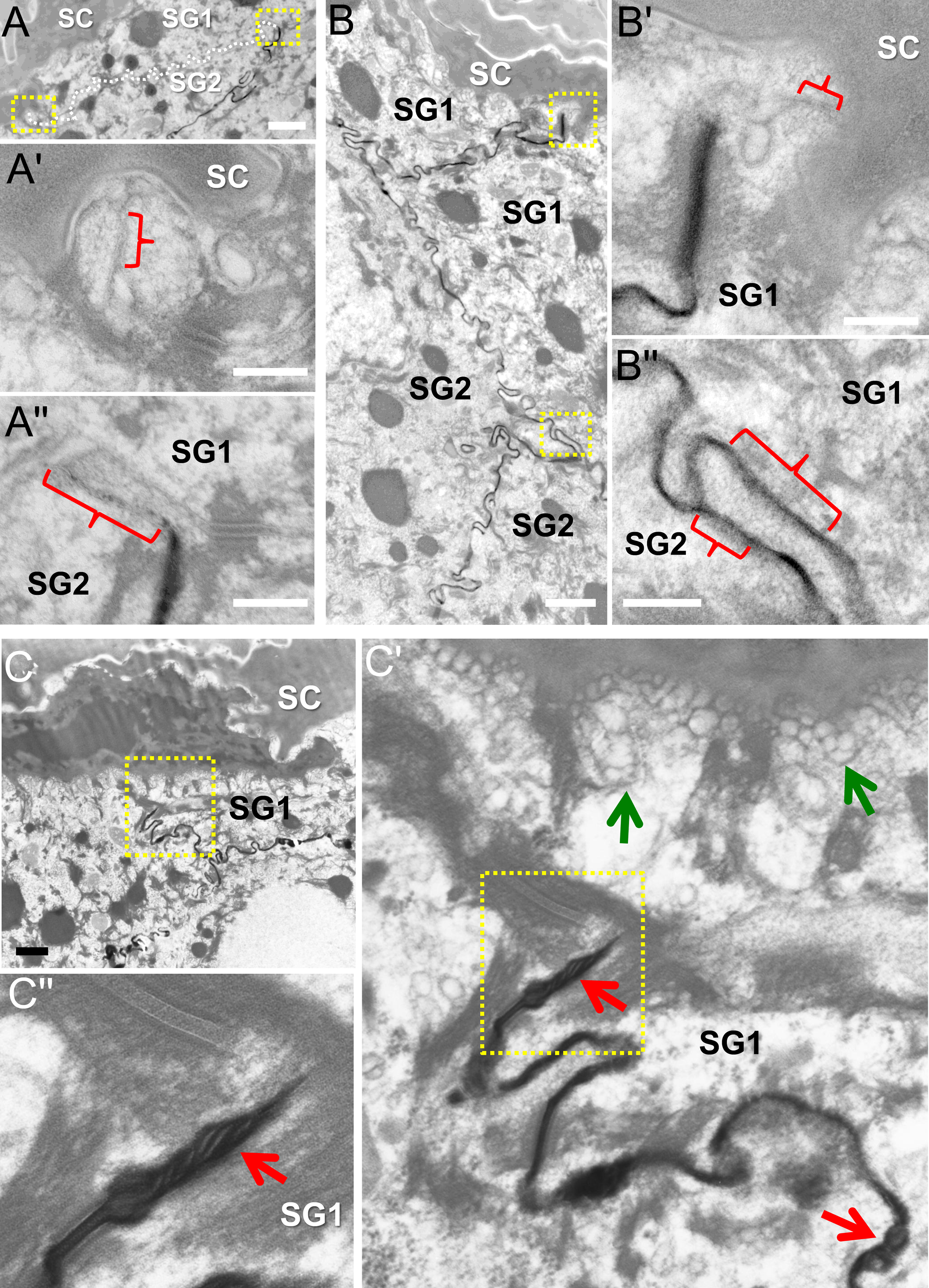

Supplement: Figure S3 — Lanthanum is blocked at the SG1 or SG2 layer and LGs can be secreted into the tracer-permeable intercellular space. A lanthanum penetration assay on newborn mouse skin. (A, B) Lanthanum is blocked at a TJ (brackets) in SG2 (A″) or passed another TJ in SG2 (B″), but stopped at a TJ in SG1 (B′). The lower border of a cell in SG1 is highlighted by a dotted line in A. (C) Most LGs are secreted from the apical domain of the SG1 (green arrows). Some were secreted into the lanthanum containing intercellular spaces (red arrows). A′, A″, B′, B″, C′ and C″ are high-magnification views of A, B, C and C′. Bars = 1 µm (A, B, C) and 200 nm (the others). (TIF) [file pone.0031641.s003.tif]

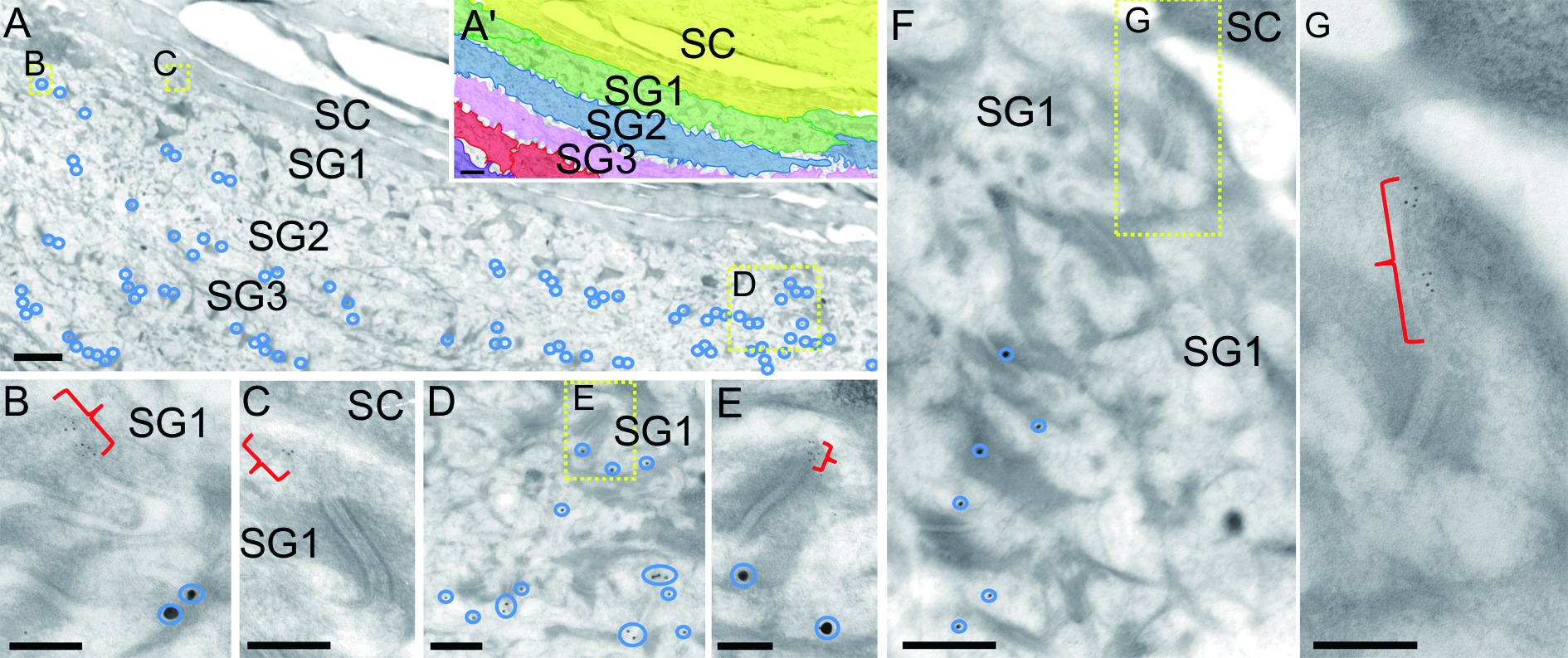

Supplement: Figure S4 — A biotin tracer is detected below the level of occludin-positive TJs in SG1. This figure is the result of the paracellular permeability assay using a biotin tracer performed on normal human skin. (A) Larger dots are silver intensified streptavidin/gold particles reacted with biotin (blue circles). The tracer is detected up to SG1. (A′) The same field as in panel A, but each stratum is indicated with different colours. B, C and D are high-magnification views of A as indicated. E is a high-magnification view of D. Occludin-positive TJs are marked with red brackets. (F) The tracer is detected below the level of occludin-positive TJ in SG1, but not in the level above it. G is a high-magnification view of F. Bars = 2 µm (A, A′), 200 nm (B, C, E, G) and 500 nm (D, F). (TIF) [file pone.0031641.s004.tif]

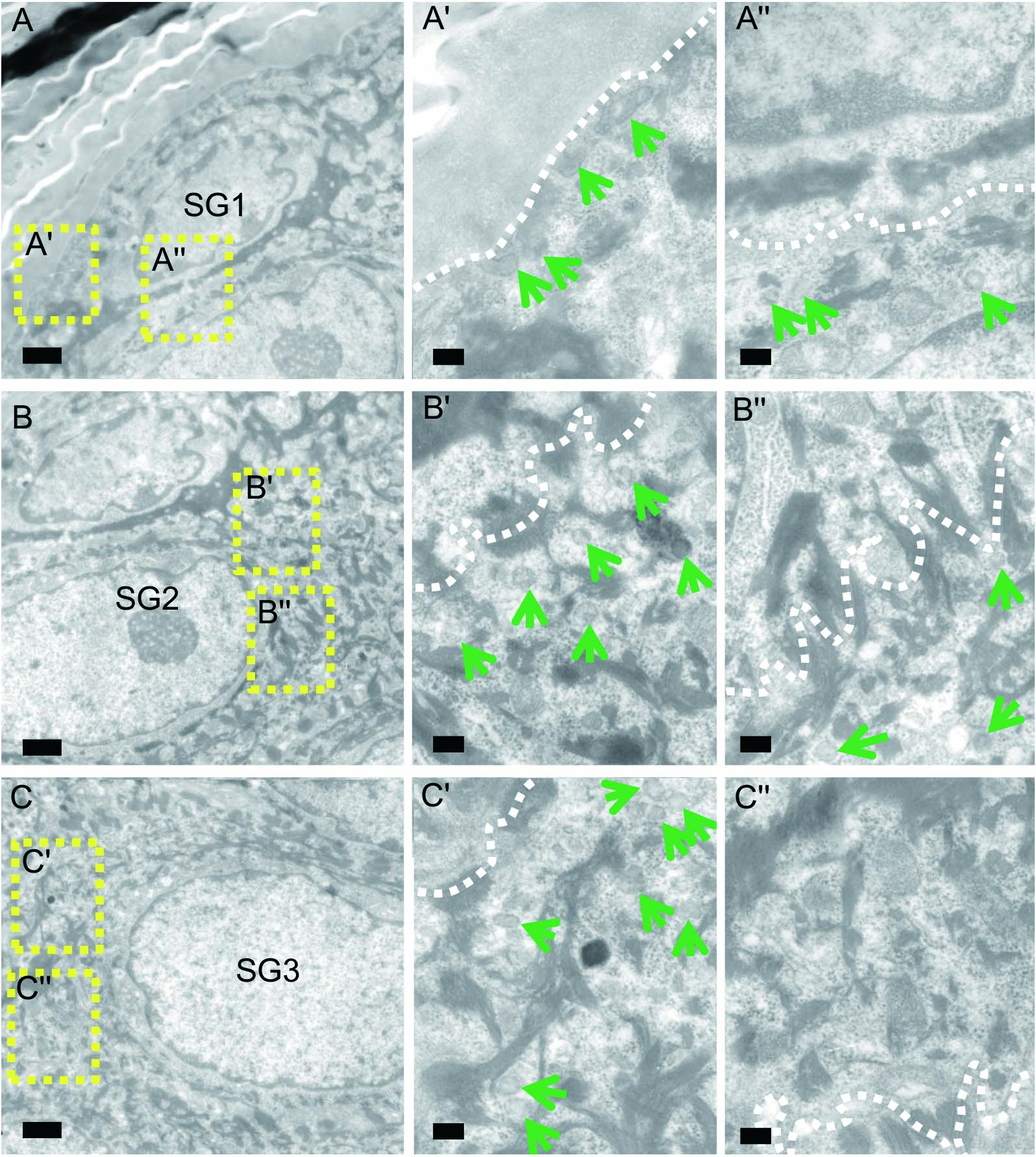

Supplement: Figure S5 — EM features indicating polarized LG localization in the SG1, SG2 and SG3. EM of normal human skin. The middle and the right columns are high-magnification views of the left column showing the apical and basal parts of the cells, respectively. Arrows, LGs. The cell borders are highlighted by dotted lines. Bars = 1 µm (A, B, C) and 200 nm (the others). (JPG) [file pone.0031641.s005.jpg]
